# Deep learning based buck-boost converter for PV modules

**DOI:** 10.1016/j.heliyon.2024.e27405

**Published:** 2024-03-05

**Authors:** Aoun Muhammad, Asjad Amin, Muhammad Ali Qureshi, Abdul Rauf Bhatti, Muhammad Mahmood Ali

**Affiliations:** a Dept. of Electrical Engineering, The Islamia University of Bahawalpur, Pakistan; b Dept. of Information & Communication Engineering, The Islamia University of Bahawalpur, Pakistan; c Dept. of Electrical Engineering & Technology, Govt. College University, Faisalabad, Pakistan; d Centre for Mathematical Modelling and Intelligent Systems for Health and Environment (MISHE), Atlantic Technological University Sligo, Ash Lane, F91 YW50 Sligo, Ireland; e Department of Mechatronic Engineering, Atlantic Technological University Sligo, Ash Lane, F91 YW50 Sligo, Ireland

**Keywords:** Photovoltaics (PV), Buck-boost converter, Parameters for stability, Proportional integral derivative (PID) controller

## Abstract

Over the past few years, the use of DC-DC buck-boost converters for Photovoltaic (PV) in renewable energy applications has increased for better results. One of the main issues with this type of converter is that output voltage is achieved with the undesired ripples. Many models are available in the literature to address this issue, but very limited work is available that achieves the desired goal using deep learning-based models. Whenever it comes to the PV, then it is further limited. Here, a deep learning-based model is proposed to reduce the steady-state time and achieve the desired buck- or boost mode for PV modules. The deep learning-based model is trained using data collected from the conventional PID controller. The output voltage of the experimental setup is 12V while the input voltage from the PV modules is 10V (when the sunlight decreases) to 24V (for 3.6 kVA) to 48V (for more than 5 kVA). It is among the few models using a single big battery (12V) for off-grid and on-grid for a single building. Experimental results are validated using objective measures. The proposed model outperforms the conventional PID controller-based buck-boost converters. The results clearly show improved performance in terms of steady-state and less overshoot.

## 1 Introduction

The converters play an important role whenever DC-to-DC conversion is required. The major types of converters are a buck [1], [2], [3], boost [4], [5], [6] and buck-boost converters [7], [8], [9]. Buck-boost converters are used when both step-up and step-down operations are required. Different types of converters depend on several components, topology, control techniques, etc. DC-DC buck-boost converters come with different applications and topologies according to the requirement. Usually, the practical converter topology used in PV applications is either buck or boost. However, there are some applications where converter topology is buck-boost [10], [11], [12]. A dual function of a buck-boost converter in a PV system with a Maximum Power Point Tracker (MPPT) and I-V tracer was proposed for fault diagnosis applications [13]. A partial buck-boost resonant power converter was proposed for residential PV applications in [14]. For grid-connected PV systems, a single-stage doubly grounded transformerless inverter topology with buck-boost voltage capability was proposed in [15]. A machine learning-based voltage regulation method was proposed using a smart rotating magnetic inverter in [16]. Using a photo-diode sun sensor, an MPPT and Artificial Neural Network (ANN)-based solar tracking system was proposed in [17].

Despite the advantages of the mentioned buck-boost converters, almost all of them have ripples in the output [18], [19], [20], [21]. The ripple problem occurs when the switching is in the process, whether there is one switch or several switches [22], [23]. The fact is that with the increase in the number of switches, the output ripple problem becomes severe. The output ripple problem is a topic of research interest nowadays. The major cause of this problem is the non-linearity in the converters produces faults, decreasing the system's overall efficiency [24], [25], [26]. Power electronics use approximation to overcome the issue. So, it was the need of the hour that deep learning was used to minimize the non-linearity.

Further, the control of the output voltage, which is a challenging nonlinear problem, might be reduced by deep learning. Convolutional Neural Network (CNN) belongs to deep learning that learns to accomplish a task by creating maximum hidden layers and at the final step adding regression layer [27], [28], [29], [30]. The main objective of deep learning is to teach computers to do what comes naturally to humans and automatically extract the features that are done manually in the case of machine learning [31], [32].

The optimization control techniques perform better than the conventional control techniques for the controller of the PV system. Two of the techniques are used in [33] to prove the effectiveness of the optimization control techniques. Full optimization is required when the PV system is under dynamic partial shading patterns. A relatively new optimization algorithm, Emperor Penguin Optimizer (EPO), has been developed [34]. Another new optimization method called Cuttlefish Algorithm (CFA) for partial shading is proposed in [35] to enhance the performance of the PV system.

In this paper, we present a deep learning-based workflow for conventional buck-boost converters to efficiently manage the system efficiency by reducing overshoot and settling time. The proposed model collects the training data using the PID controller attached to the buck-boost converter. The data is then used with the deep learning-based model. The trained model used with the buck-boost converter produces the desired buck and boost levels with reduced overshoot.

The rest of the paper is organized as follows. Section 2 discusses the methodology, including controller and deep learning. Section 3 discusses the buck-boost converter with its brief work. Experimental results are presented in Section 4. Finally, the conclusion is discussed in Section 5.

## 2 Methodology

**Figure 1:**
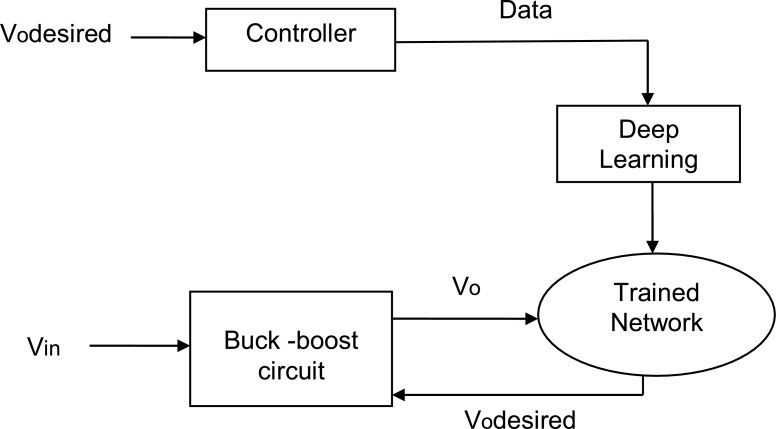
Block diagram of the proposed deep learning framework used for training.

The desired output value $V_{o,desired}$ is provided to the controller, which provides the data to the deep-learning network. Deep learning is then used as the network model. Conversely, the input voltage $V_{in}$ is provided to the buck-boost circuit, and the output voltage $V_{o}$ is obtained as an output. The output voltage $V_{o}$ from the buck-boost circuit is provided to the trained network. The trained network then provides $V_{o,desired}$ to the buck-boost converter, which provides the output voltage to the trained network and continues until the output voltage is without ripples. Fig. 1 shows the workflow of the proposed model using a deep learning framework.

### 2.1 Controller

Usually, linear control techniques are used in which Proportional Integral and Derivative (PID) controllers are popular. For constant output value, tuning derivative, proportional, and integral constants is necessary to obtain the stable output value. However, the buck-boost converter's nonlinearity makes linear control techniques less efficient. PI-like fuzzy controllers have also provided better results compared to conventional controllers. There are some hybrid algorithms available for that purpose.

### 2.2 Deep learning

Deep learning is used in power electronics and has shown promising results in optimizing power conversion systems and improving their reliability. The major challenge in power electronics is to deal with electrical stresses and instabilities, which can lead to system failure or reduced efficiency. Deep learning algorithms can be trained on large data sets to recognize patterns and predict these events before they occur, allowing for preventative maintenance and improved system performance. The proposed model's training process considered electrical stresses and instabilities in terms of input and output voltage changing with different combinations. By training deep neural networks on large data sets of power electronics system behavior, these networks can learn to identify patterns associated with particular faults or instabilities, help diagnose early problems, and prevent system failure.

Deep learning can also be used for predictive maintenance in power electronics systems. Monitoring the system's behavior over time and training deep neural networks on this data makes it possible to predict when components may fail or when maintenance is needed before it becomes an issue that affects system performance.

With time, machine learning approaches were used with the converters to get better results compared with the conventional methods. Krishnamoorthy et al. in [36] proposed machine learning-based power electronic converters modeling. Fu et al. in [37] proposed a data-driven framework using support vector machine, expectation maximization, and principal component analysis techniques for fault classification in non-inverting buck-boost DC-DC power converters. Abegaz et al. in [38] proposed a method for dynamic switching control of buck converters using unsupervised machine learning. Wu et al. in [39] developed a high step-up/step-down three-port bidirectional DC/DC converter for photovoltaic systems. In the earlier applications, machine learning with buck-boost converters was introduced, and buck-boost converters for PV module applications were discussed [40], [41]. However, the use of machine learning, especially deep learning with buck-boost for PV modules in the literature, is very limited [42], [30], [43]. This work proposes a deep learning-based controller for a buck-boost converter for PV module applications.

**Figure 2:**
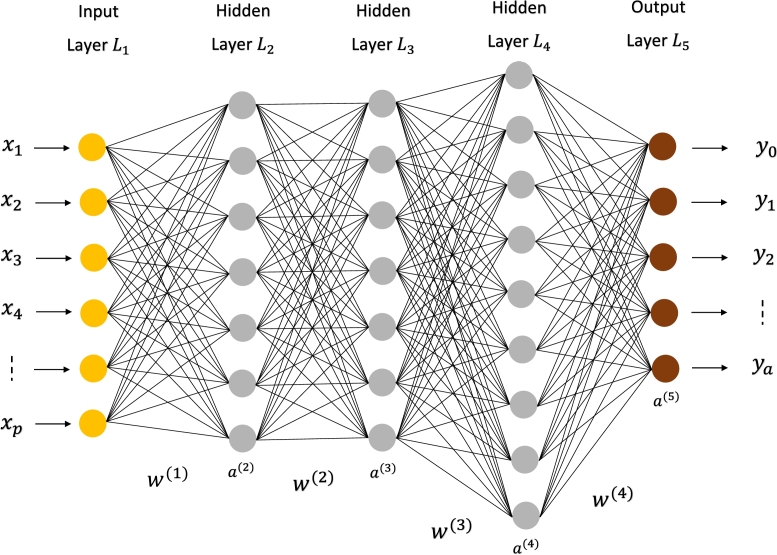
Block diagram of a typical deep learning network for regression.

Deep learning is used to train machines like humans do, i.e., learning by example. Here, classification is achieved by learning directly from text, sound, images, and/or videos. Deep learning models result in high accuracy and generally exceed human beings. Deep learning methods are also called deep neural networks, as they mostly use neural network architectures. In deep networks, the keyword “deep” refers to large numbers of hidden layers. Traditional neural networks usually have 2–3 hidden layers while the number of hidden layers in deep neural networks exceeds 100 [44], [45]. Deep learning models require large sets of labeled data for training and do not require hand-crafted features for learning, they directly learn from the data itself. Fig. 2 shows the framework of a typical deep-learning network. Here, the input vector *X* will be replaced by the input voltage $V_{in}$, and the output vector *Y* will be the desired output voltage $V_{o,desired}$.

Tong et al. in [46] used deep learning to diagnose modular multilevel converter (MMC) faults. Similarly, Yu et al. in [47] used deep learning for adaptive steady-state modeling and fast control strategy for CLLC DC-DC converter in renewable penetrated systems. One of the most popular types of deep neural networks is the Convolutional Neural Network (CNN). A CNN convolves learned features with input data and uses 2D convolutional layers. As in our case, a regression layer may be the last for regression purposes. In machine learning, feature extraction is manual, while feature extraction is automatic in deep learning.

## 3 Buck-boost converter

**Figure 3:**
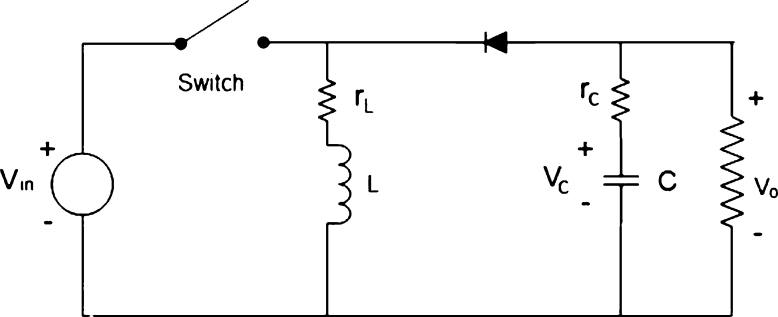
Buck-boost converter in open loop.

**Figure 4:**
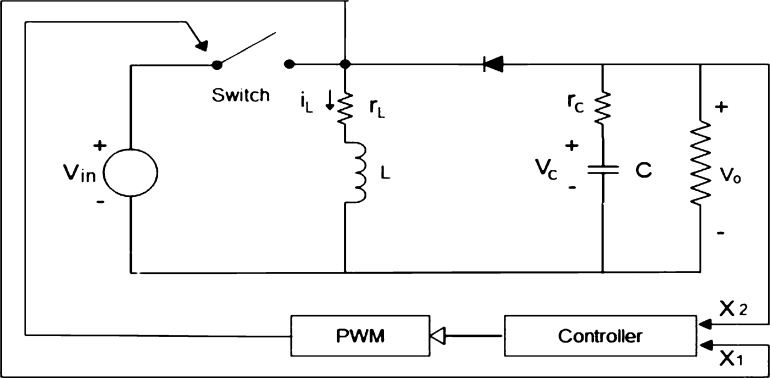
Buck-boost converter in closed loop.

In this work, we aim to propose a deep learning-based buck-boost converter for PV applications. The proposed model combines a conventional buck-boost converter and ANN in this work. Therefore, it is necessary to provide the details of the buck-boost converter's state-space model to further elaborate on the conventional converter. Buck-boost converters in an open loop (without feedback control) and in a closed loop (with feedback control) are shown in Figure 3, Figure 4 respectively. Two linear state-space models are used to model the behavior of the buck-boost converter when the converter switch is ON and OFF state. When the converter switch is ON, the inductor stores energy in its magnetic field, and when the switch is OFF, the magnetic field is de-energized to maintain the current flow through the load. The load voltage is almost the same as that of the inductor voltage. The dynamic response and regulation characteristics are not good for the buck-boost converter in open-loop mode. Therefore, it is always recommended to use it in the closed loop. To maintain a constant output voltage, i.e., 12V, the controller changes the duty cycle of the pulse width modulator with the present state of the buck-boost converter. The buck-boost converter works in buck or boost mode depending on the duty cycle. In buck mode, it indeed steps down the input voltage to a lower value and steps up the input voltage to a higher value during boost mode based on the duty cycle.

Where

$V_{in}$ = input voltage or supply voltage

*L* = inductor

$r_{L}$ = internal resistance of the inductor

*C* = capacitor

$r_{c}$ = internal resistance of the capacitor

$V_{c}$ = voltage across capacitor

Switch = MOSFET

$V_{o}$ = output voltage

$i_{L}$ = inductor current

$R_{L}$ = load resistance (resistance along the output voltage).

$X_{1}=i_{L}$ = inductor current

$X_{2}=V_{c}$ = capacitor voltage

$V_{in}=V_{G}$

$r_{m}$ = resistance of the switch

$r_{d}$ = resistance of the diode

$V_{D}$ = voltage across the diode

$V_{M}$ = voltage across the switch

The state-space equations help design and analysis of the model. So, the state-space equations for the proposed model in the “ON” and “OFF” states are derived from Eqs. (1)–(11).

(1) $$\left\{ \begin{array}{c} \overset{˙}{x}=A_{1}\;x+B_{1\;}u \end{array}\right.$$

(2) $$x=\left[\begin{array}{c} i_{L}\\ v_{c} \end{array}\right]u=\left[\begin{array}{c} v_{G}\\ i_{O}\\ v_{M}\\ v_{D} \end{array}\right]$$

(3) $$A_{1}=\left[\begin{array}{cc} \frac{-(r_{L}+r_{m})}{L}\; & 0\\ 0\; & \frac{-1}{(R+r_{c})C} \end{array}\right]$$

(4) $$B_{1}=\left[\begin{array}{cccc} \frac{1}{L}\; & 0\; & \frac{-1}{L}\; & 0\\ 0\; & \frac{-R}{(R+r_{c})C}\; & 0\; & 0 \end{array}\right]$$

(5) $$\left\{ \begin{array}{c} \overset{˙}{x}=A_{2}\;x+B_{2\;}u \end{array}\right.$$

(6) $$x=\left[\begin{array}{c} i_{L}\\ v_{c} \end{array}\right]u=\left[\begin{array}{c} v_{G}\\ i_{O}\\ v_{M}\\ v_{D} \end{array}\right]$$

(7) $$A_{2}=\left[\begin{array}{cc} -\frac{Rr_{c}+Rr_{L}+r_{L}r_{c}+Rr_{d}+r_{d}r_{c}}{L(R+r_{c})}\; & \frac{-R}{L(R+r_{c})}\\ \frac{R}{(R+r_{c})C}\; & \frac{-1}{(R+r_{c})C} \end{array}\right]$$

(8) $$B_{2}=\left[\begin{array}{cccc} 0\; & \frac{Rr_{c}}{\left(R+r_{c}\right)L}\; & 0\; & \frac{-1}{L}\\ 0\; & \frac{-R}{\left(R+r_{c}\right)C}\; & 0\; & 0 \end{array}\right]$$

(9) $$\left\{ \begin{array}{c} \overset{˙}{x}=A_{P}\;x+B_{P\;}u \end{array}\right.\left\{ \begin{array}{c} A_{P}=A_{1}d+A_{2}(1-d)\\ B_{P}=B_{1}d+B_{2}(1-d) \end{array}\right.$$

(10) $$A_{P}=\left[\begin{array}{cc} -\frac{-\left(r_{L}+r_{m}\right)\left(R+r_{c}\right)+(r_{m}-r_{d})(R+r_{c})d^{\prime }-Rr_{c}d^{\prime }}{L(R+r_{c})}\; & \frac{-Rd^{\prime }}{L(R+r_{c})}\\ \frac{Rd^{\prime }}{(R+r_{c})C}\; & \frac{-1}{(R+r_{c})C} \end{array}\right]$$

(11) $$B_{P}=\left[\begin{array}{cccc} \frac{1-d^{\prime }}{L}\; & \frac{Rr_{c}d^{\prime }}{(R+r_{c})L}\; & \frac{-1+d^{\prime }}{L}\; & \frac{-d^{\prime }}{L}\\ 0\; & \frac{-R}{(R+r_{c})C}\; & 0\; & 0 \end{array}\right]$$

### 3.1 Deep-learning based buck-boost converter model

**Figure 5:**
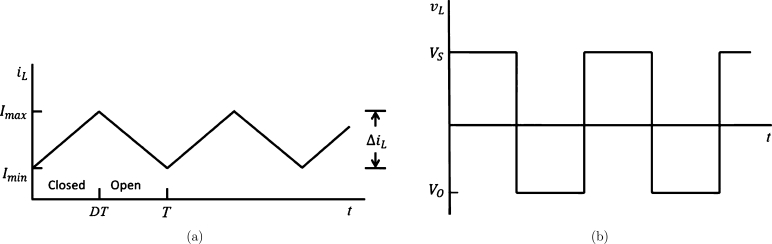
Buck-boost converter waveforms of (a) inductor current (b) inductor voltage.

The trained deep learning-based model is used with the conventional buck-boost converter for PV module applications. In this paper, we consider $V_{i}$ = 10V as input voltage means the solar input is low to the case when the solar system is with 3 kVA and 24V to more than 5 kVA with 48V. The advantage of this model is that up to a 10 kVA system, a single battery with large storage can be replaced by replacing the current system, i.e., two for mid-range 3 kVA to 5 kVA or more up to 10 kVA with four batteries. Fig. 5(a) and (b) show voltage and current waveforms respectively, for the ideal buck-boost converter. Here, $R_{L}$ and $V_{L}$ are the inductor resistance and voltage drop respectively, across the inductor, *L*. Our primary goal is to control the average current value flowing through the inductor. As average values of inductor current and voltage are duty cycle dependent, therefore, we used it in our design model.

## 4 Results and discussions

In this paper, the proposed deep learning-based buck-boost converter is validated using both simulation and hardware. The model is learned using the training data acquired from the conventional PID controller. The samples are taken at the rate of 50 samples per level corresponding to D=[0.20,0.25,0.33,0.54]. The input voltages are $V_{in}=10$V, 24V, 36V, and 48V and the output voltage is $V_{o}=12$V.

### 4.1 Simulation results

**Figure 6:**
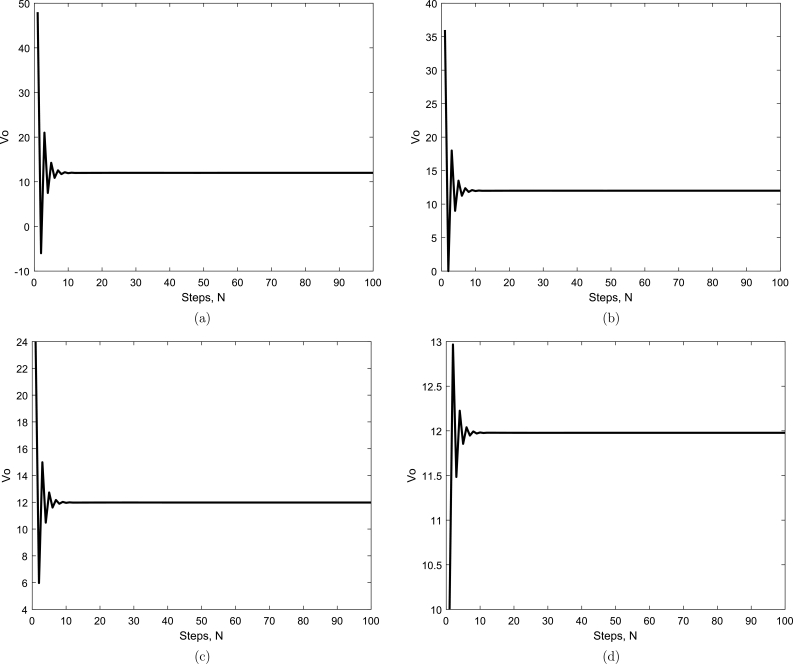
PID controller output voltage *V*_*o*_ with different values of duty cycles (a) *D* = 0.20, (b) *D* = 0.25, (c) *D* = 0.33, (d) *D* = 0.54.

Here, we compare output voltages with different values of duty cycles for conventional PID controllers and deep learning-based controllers. The graphs of output voltage $V_{o}$ with different values of duty cycle, *D*, by using PID controller are shown in Figs. 6(a to d). By observing the graphs in Fig. 6(a) and (b), there is a lot of oscillation initially around amplitude of 10V to 24V peak to peak with duty cycles 0.20 and 0.25, during the transient condition and that overshoot is not too high. Comparing the graphs of Figs. 6(c) and (d) to the graphs of Figs. 6(a) and (b), there is oscillation with an amplitude of 36V to 60V peak to peak with duty cycles 0.33 and 0.54 which is high compared to the previous graphs. Still, there is high overshoot for all the cases. Considering all the next graphs of Figs. 6(a) to 6(d) with duty cycles 0.2 to 0.54, it is obvious that during the transient condition, there is a lot of oscillation initially around the set-point with amplitude too high. The overshoot is too high during all graphs of Fig. 6, and that overshoot can not be neglected. All the responses in the graphs in Fig. 6 show a high settling time that makes the buck-boost converter slow by decreasing the overall efficiency.

**Figure 7:**
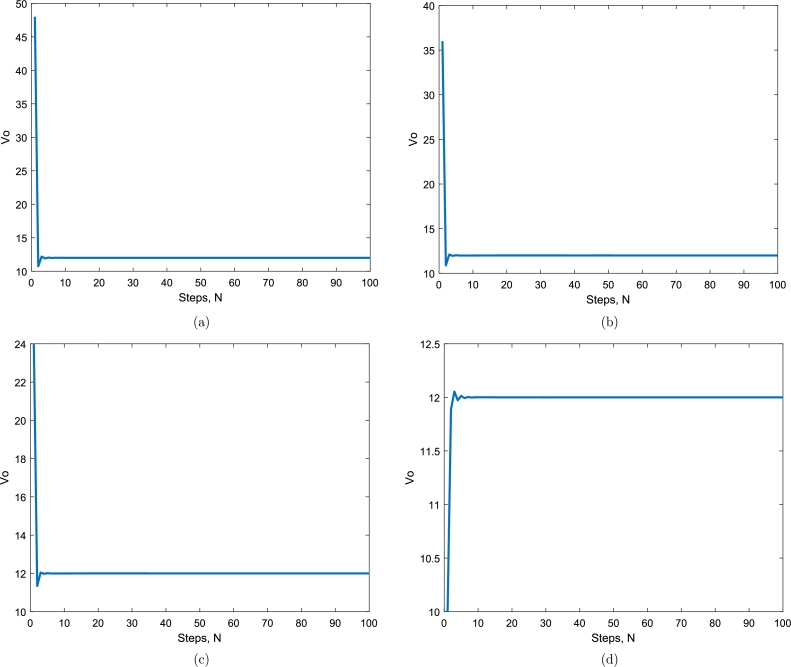
Deep Learning output voltage *V*_*o*_ for simulation results with different values of duty cycles (a) *D* = 0.20, (b) *D* = 0.25, (c) *D* = 0.33, (d) *D* = 0.54.

The graphs of output voltage $V_{o}$ with different values of duty cycle, *D*, by deep learning are shown in Figs. 7(a to d). By observing both the graphs in Figs. 7(a) and (b), there is no oscillation or minor oscillation with an amplitude of 0.1V peak to peak with duty cycles 0.20 and 0.25 during the transient condition and there is almost no overshoot at all. Comparing the next graph of Figs. 7(c) and (d) to the previous graphs, there is a little bit of oscillation of 1V amplitude during the transient condition, and the overshoot is almost nil compared to the PID controller graph in Fig. 6 of the same duty cycle. The graphs in Figs. 7(a) to (d) demonstrate that there is very negligible oscillation with an average amplitude of 1V to 2V under transient conditions. The overshoot is too low and may be neglected. All the responses in the graphs in Fig. 7 show a very low settling time, making the buck-boost converter very fast by increasing overall efficiency.

### 4.2 Experimental results

**Figure 8:**
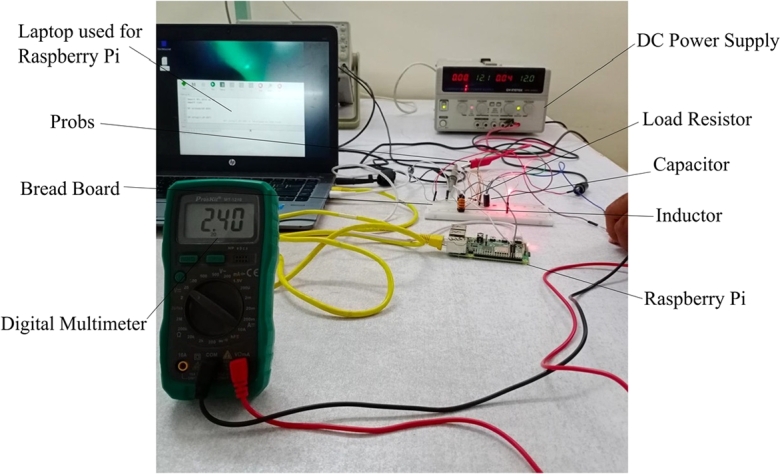
Experimental prototype setup.

**Figure 9:**
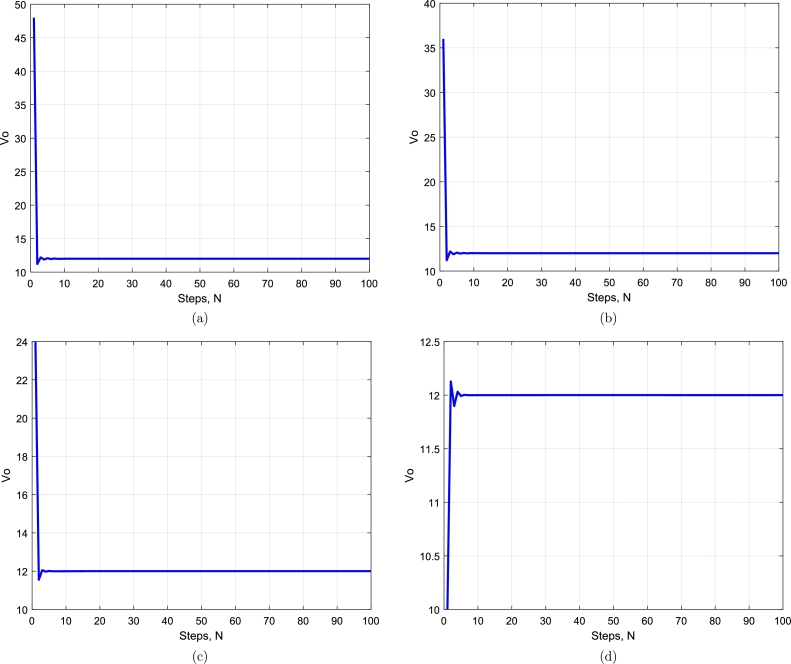
Deep learning output voltage *V*_*o*_ for experimental results with different values of duty cycles (a) *D* = 0.20, (b) *D* = 0.25, (c) *D* = 0.33, (d) *D* = 0.54.

This paper compares the simulation and hardware results of the proposed deep learning-based method with the conventional PID controller. The proposed converter uses N-channel power MOSFET and is powered by a programmable power supply. Raspberry Pi-4TM controller is used for gating pulse and an isolation circuit is used for the deep learning algorithm. Raspberry Pi-4TM controller is selected based on computation power and characteristics from the manufacturer's datasheet. GW INSTEK GDS-1102-U hardware instrument is used to capture the voltage waveforms. The hardware prototype of the proposed buck-boost converter is shown in Fig. 8. A load resistor at the output of the buck-boost converter is used for power dissipation. The isolation circuit brings the voltage level of the converter for efficient operation in a closed loop. Figure 7, Figure 9 show the simulation and experimental results regarding output voltage waveforms for the deep learning-based buck-boost converter, respectively. It is important to mention that the system is indeed for 3 kVA to 10 kVA. As for the initial system, one battery is required with 12V, then two batteries are required with 24V, and finally, for higher kVA, four batteries are required with 48V. The experimental results are considered for the four values of the duty cycle, which correspond to the inputs usually used in PV modules. All these voltage levels are tested for the battery requirements and working point of view. A summary of system parameters and components used in the experiment setup is provided in Table 1.

**Table 1:** Summary of system parameters and components used in the experiment setup.

Component/Parameter	Values
Pulse generator frequency	100000*Hz*
Inductor (*L*)	275*μH*
Capacitor (*C*)	0.551μF
Load Resistor (*R*_*o*_)	5*k*Ω
Input Voltage (*V*_*in*_)	12V, 24V, 36V, 48 V
Output Voltage (*V*_*o*_)	12V

It is obvious from Figs. 7(a) and 9(a) that when the duty cycle D=0.20, the input voltage is 10V and the output is more stable using deep-learning as compared to PID controller results in Fig. 6. The converter is working in boost mode, and the results are close to experimental results. It is obvious from the above Figs. 7(b) and 9(b) that when the duty cycle D=0.25, the input voltage is 24V and the output is more stable using deep-learning as compared to PID controller results in Fig. 6 and the converter is working in buck mode, and the results are close to experimental results. A similar comparison comes true for Figs. 7(c) and 9(c), when the duty cycle is D=0.33, the input voltage is 36V and the output is more stable using deep learning compared to PID controller results in Fig. 6 and the mode of the converter is the buck verified by experimental results. Considering duty cycle D=0.54, when Figs. 7(d) and 9(d) are compared for the input voltage 48V, and the output of deep-learning becomes stable very early as compared to PID controller output in Fig. 6(d) and the converter is working in buck mode with the experimental results verified.

**Figure 10:**
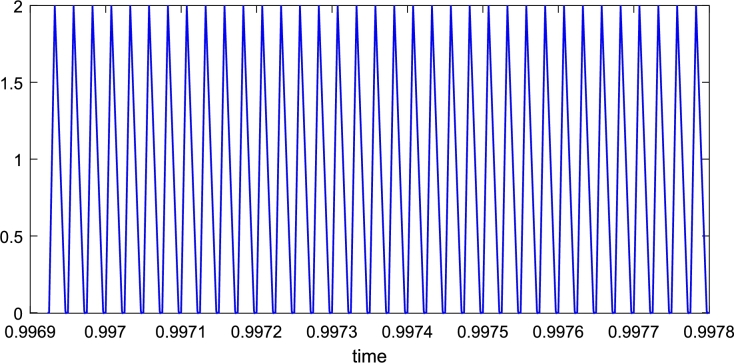
A current waveform validating the behavior of the inductor.

For our proposed deep learning-based model, experimental results are very close to the simulated results regarding overshoot and much better than the PID controller results shown in Fig. 6. Similarly, the experimental results are the same or very close to the simulated results regarding settling time and much better than the PID controller. There is hardware uncertainty in the results, as shown in Fig. 9 compared with the simulated results in Fig. 7. These uncertainties in hardware, e.g., in capacitor and inductor that were not considered for simulation results make the experimental results minor change compared to the simulation results. The current waveform validating the behavior of the inductor is shown in Fig. 10.

From the results, we observe that experimental hardware results are very close to the simulation results. This is due to the reason that many parameters and load variation losses are incorporated into the deep learning algorithm. Therefore, it is concluded that deep-learning results become stable earlier than the results using the PID controller.

### 4.3 Performance analysis

The achievement of the steady state of the proposed model is analyzed using the existing state-of-the-art evaluation measures in the literature. These evaluation measures are discussed and analyzed for the proposed model.

#### 4.3.1 Normalized overshoot

The normalized overshoot can be calculated as follows:

(12) $$\text{Normalized overshoot}=\frac{Y_{m}-Y_{desired}}{Y_{desired}}$$

where $Y_{m}$ is the maximum value of the signal along the y-axis. It is the maximum voltage overshoot or undershoot height along the y-axis. Another term that is not too popular as “overshoot” but is discussed in the literature is “undershoot”. The occurrence of a signal or function lower than the target value may be termed an undershoot. Here, it covers both overshoots and undershoots. $Y_{desired}$ is the desired value of the voltage.

#### 4.3.2 Settling time

The settling time is when overshoot or undershoot takes for the output to reach the steady-state value. The achievement of the steady state of the proposed model using objective measures is presented in Table 2. If the value of normalized overshoot or undershoot is more, then the achievement of the steady-state is less and vice versa. If the value of settling time is less, then it means that the overshoot or undershoot exists for very less time. In other words, the system achieves steady-state earlier and vice versa. Now, in Table 2, there are three types of values calculated for normalized overshoot and settling time. These are for PID controller, proposed Deep-learning-based model simulated results, and proposed Deep-learning-based model hardware results. It can be observed from the table that the numerical values of normalized overshoot for PID controller are very high as compared to the values of proposed Deep-learning based model simulated results and the value is too high when the duty cycle is D=0.2. Similarly, the settling time of the proposed Ensemble-learning-based simulated results for all duty cycle values is very low compared to the PID controller.

**Table 2:** Objective Evaluation Measures.

Duty cycle	Controller	Normalized overshoot	Settling time
0.2000	PID	1.5100	0.2200
	Proposed simulated	0.0266	0.1602
	Proposed hardware	0.0223	0.3903

0.2500	PID	0.6668	0.2100
	Proposed simulated	0.0280	0.1802
	Proposed hardware	0.0595	0.2003

0.3333	PID	0.2501	0.1910
	Proposed simulated	0.0627	0.1210
	Proposed hardware	0.0627	0.1410

0.5454	PID	0.1670	0.1810
	Proposed simulated	0.0196	0.1210
	Proposed hardware	0.0225	0.1650

When the values for normalized overshoot of PID controller are compared with the values of proposed Deep-learning based model hardware results, it is obvious that these are high for PID controller as compared to the proposed Deep-learning based model hardware results. When the values of the proposed Deep-learning model simulated results are compared with the hardware results, the hardware results are the same or very close to the simulated results for normalized overshoot. A slight variation is due to the hardware losses and their effects. The PID controller's settling time is higher than the proposed Ensemble-learning-based model hardware results. The values of the proposed Deep-learning-based model simulated results are very similar to the hardware results.

It is concluded that the settling time of the proposed Deep-learning-based model hardware results is very less as compared to the PID controller, showing the earlier achievement of steady-state of the proposed model compared to the PID controller in terms of settling time. Similarly, the normalized overshoot of the proposed Deep-learning model is less compared to the PID controller. The proposed deep learning-based converter achieves steady-state earlier than the PID controller and has a better achievement of steady-state results validated by the existing objective evaluation measures.

Table 2 clearly shows that the percentage overshoot of the proposed model is of very less value as compared to the PID controller for all values of the duty cycle. The settling time of the proposed model is much less compared to the PID controller for all values of the duty cycle. In short, all the objective measures show that the Deep-learning-based buck-boost controller is much better than the PID-based controller model. In the proposed work, the system is trained for the dynamic response for the supply voltage or input voltage and desired output voltage with different values but with little emphasis on the load variation because the model's target is the supply voltage and desired output voltage.

The proposed deep learning-based model is accurate both in its dynamic response (for simulation results) and steady-state response (for experimental setup). Although applying deep learning in photovoltaic converter topology control is computationally expensive, in our case, the computational cost is lowered because the model automatically detects the set point when the steady state is reached.

## 5 Conclusion

In this paper, a Deep learning-based model is developed for PV module applications using the buck-boost converter to deal with the output steady-state issue. The model is trained using the data from the conventional PID controller. The trained model used with the Buck-Boost converter improves the output voltage steady-state achievement compared to the traditional PID controller. The model is successfully tested and the experimental results verify that the model developed using a deep-learning-based model provides results achieving steady-state earlier compared to the PID controller. The output voltage of the experimental setup is 12V, while the input voltage varies from (10V to 48V) suitable for (0.6 kVA to 10 kVA). The achievement of the earlier steady-state of the model is verified by the numerical values of normalized overshoot and settling time. There is a reduction in overshoot in terms of the objective measures discussed. It verifies the superior performance of the proposed model compared to the conventional PID controller. It is worth mentioning that the dataset developed in this work comprises a limited number of samples that may compromise the performance of deep learning-based models. An extension of the dataset with more samples is recommended for future work.

## CRediT authorship contribution statement

**Aoun Muhammad:** Writing – review & editing, Writing – original draft, Methodology, Data curation, Conceptualization. **Asjad Amin:** Writing – review & editing, Writing – original draft, Supervision, Conceptualization. **Muhammad Ali Qureshi:** Writing – review & editing, Writing – original draft, Supervision, Methodology, Data curation. **Abdul Rauf Bhatti:** Writing – review & editing, Writing – original draft, Data curation. **Muhammad Mahmood Ali:** Writing – review & editing, Writing – original draft, Data curation.

## Declaration of Competing Interest

The authors declare that they have no known competing financial interests or personal relationships that could have appeared to influence the work reported in this paper.

## Data Availability

Data will be made available on request.
